# Dynamical Complexity of the 2015 St. Patrick’s Day Magnetic Storm at Swarm Altitudes Using Entropy Measures

**DOI:** 10.3390/e22050574

**Published:** 2020-05-19

**Authors:** Constantinos Papadimitriou, Georgios Balasis, Adamantia Zoe Boutsi, Ioannis A. Daglis, Omiros Giannakis, Anastasios Anastasiadis, Paola De Michelis, Giuseppe Consolini

**Affiliations:** 1 Institute for Astronomy, Astrophysics, Space Applications and Remote Sensing, National Observatory of Athens, Metaxa and Vas. Pavlou St., Penteli, 15236 Athens, Greece; constantinos@noa.gr (C.P.); zboutsi@noa.gr (A.Z.B.); iadaglis@phys.uoa.gr (I.A.D.); og@noa.gr (O.G.); anastasi@noa.gr (A.A.); 2 Space Applications & Research Consultancy, SPARC P.C., 10551 Athens, Greece; 3 Department of Physics, National and Kapodistrian University of Athens, Panepistimiopolis, Zografos, 15784 Athens, Greece; 4 Hellenic Space Center, 15231 Athens, Greece; 5 Istituto Nazionale di Geofisica e Vulcanologia, 00143 Rome, Italy; paola.demichelis@ingv.it; 6 INAF-Istituto di Astrofisica e Planetologia Spaziali, 00133 Rome, Italy; giuseppe.consolini@iaps.inaf.it

**Keywords:** dynamical complexity, entropy, magnetic storm, space weather, Swarm mission

## Abstract

The continuously expanding toolbox of nonlinear time series analysis techniques has recently highlighted the importance of dynamical complexity to understand the behavior of the complex solar wind–magnetosphere–ionosphere–thermosphere coupling system and its components. Here, we apply new such approaches, mainly a series of entropy methods to the time series of the Earth’s magnetic field measured by the Swarm constellation. We show successful applications of methods, originated from information theory, to quantitatively study complexity in the dynamical response of the topside ionosphere, at Swarm altitudes, focusing on the most intense magnetic storm of solar cycle 24, that is, the St. Patrick’s Day storm, which occurred in March 2015. These entropy measures are utilized for the first time to analyze data from a low-Earth orbit (LEO) satellite mission flying in the topside ionosphere. These approaches may hold great potential for improved space weather nowcasts and forecasts.

## 1. Introduction

The Earth’s magnetosphere is a very complex real system of fields, currents and particle populations that are not only driven, but also strongly respond to solar wind dynamics, often in a highly non-linear and complicated way. The study of these non-linear processes begun since the very early 90 s, with the seminal paper by Tsurutani et al. [1] which showed the non-linear response of the Auroral Electrojet (AE) index, a geomagnetic index that monitors magnetospheric substorm activity, to the southward component of the interplanetary magnetic field (IMF) and continued by a multitude of authors [2,3,4,5] who focused on chaotic processes of low dimensionality, while others such as Klimas et al. [6] addressed the nonlinear character of magnetosphere dynamics, or Consolini et al. [7] the multifractal and turbulent nature of this dynamics, or Angelopoulos et al. [8] who focused on the plasma sheet and highlighted many aspects of its dynamical complexity. All these, coupled with the more recent advances in the study of complex systems open new research perspectives for the investigation of magnetospheric dynamics [9,10,11,12].

Detecting and quantifying changes in the dynamical complexity of complex systems from series of measurements are some of the most important problems in physics, but also in biology, engineering, and economic sciences. In all these fields, but even more in geomagnetism and magnetospheric physics, accurately discerning the differences between normal (quiet) conditions and abnormal states (when particularly energetic events take place, such as magnetic storms) can help tremendously in forecasting and consequently mitigating, space weather hazards, that affect the lifetime and behaviour of satellite systems and ground-based infrastructure.

The data used in most space physics studies are usually non-stationary, short in duration and stochastic. One of our objectives is to find an effective complexity measure that requires short data sets for statistically significant results, provides the ability to make fast and robust calculations, and can be used to analyse nonstationary and noisy data, which is convenient for the analysis of geomagnetic and magnetospheric time series. Entropy measures have been proven to be a useful tool to investigate the dynamical complexity of magnetic storms [13,14]. Moreover, time series analyses based on entropy approaches and concepts related to information transfer have been used to shed light on the magnetic storm–magnetospheric substorm relationship [15,16] and the solar wind drivers of the outer radiation belt [17].

The purpose of this paper is to demonstrate the applicability of a variety of entropy measures [for example, Shannon, Tsallis, approximate entropy (ApEn), sample entropy (SampEn) and fuzzy entropy (FuzzyEn)], already proved to be useful in the study of the Disturbance storm-time (Dst) index, a geomagnetic index that monitors magnetic storm activity (see for instance the review [18]), to determine/quantify the concept of changing system complexity, using Swarm data associated with the occurrence of the most intense magnetic storm of the solar cycle 24 (the St. Patrick’s Day storm occurred on 17 March 2015 with a minimum Dst index of −223 nT). Herein, these information-theoretic techniques are utilized for the first time to analyze data from a low-Earth orbit (LEO) satellite mission flying in the topside ionosphere. High quality Swarm observations offer a unique opportunity to understand the impact of intense magnetic storms to the magnetosphere-ionosphere-thermosphere coupling system. Furthermore, the outcome of these entropy methods could have a great potential impact in forecasting extreme space weather events and, thus, mitigating space weather hazards to technological infrastructure.

Most space physicists and space weather experts are not familiar with the entropy concepts and related analysis techniques presented herein. One of the aims of this study is to increase the degree of familiarity of Heliophysics scientists on using these information theory approaches to tackle contemporary research problems in Space Physics.

In the rest of this paper, Section 2 presents an outline of some methods that emerged from the field of Information Theory, showcasing both different mathematical formulations for the calculation of an entropy-like quantity and different approaches in the application of such measures, for example, by using the concept of symbolic dynamics, by which a digitization is performed in the range of value of a series to facilitate the search for “patterns” in the temporal sense. Section 3 shows the application of such methods to quantify the changes in the complexity of the magnetosphere-ionosphere coupling system, as it responds to the onset and evolution of the intense magnetic storm of March 2015, while Section 4 summarizes the findings and conclusions of the analysis.

## 2. Methodology

Since Ludwig Boltzmann’s statistical definition of entropy in 1877, many entropy-like measures have been defined to quantify the randomness or the degree of disorder of a wide range of systems, with applications ranging from telecommunications and cryptography [19] to environmental sciences and space physics. This section presents an outline of some of the most well-known measures and methods.

### 2.1. Shannon Entropy

In his seminal paper, back in the late 1940s, Claude Shannon [20] described a telecommunication system as a Markov process [21] and defined the entropy *H*, as a measure of the information that is being produced by it. In his formulation, which was of course based on Boltzmann’s famous *H* theorem [22], $p_{i}$ denotes the probability of the system being in a cell *i* of its phase space.

(1) $$\begin{array}{c} H=-\sum p_{i}\cdot \log p_{i}. \end{array}$$

In real world applications, one rarely has access to observables pertaining to the entire phase space that is available to a system. Instead, experimental results are often limited to the values obtained by a few number of measurable parameters. Shannon’s formulation can be easily extended to such cases, although it is necessary to discretize the range of values of the observed parameter and consider each distinct point in this new discrete space as a realization of a different cell of the system’s phase space. In this sense, the probability $p_{i}$ can be calculated by measuring the number of observations that belong to each such point and dividing by their total number. The discretization can be performed in a multitude of ways, the simplest and most common of them being the equal-width and the equal-frequency discretizations [23], the latter of which, by its definition, maximizes the Shannon Entropy.

### 2.2. Symbolic Dynamics and Block Entropy

The discretization that was discussed obviously removes a certain amount of information from the system, as its outputs are now described in terms of specific states instead of in values of a continuous variable. Despite that, this approach has the potential to provide much more important details about the dynamics of the system, if one also considers the succession of the various states and not just the statistics of their instantaneous values. In this way the trajectory of the system is now represented by sequences of numbers, each of which corresponds to a particular cell of the discretized space, exchanging the loss of information on the accuracy of each state for information on other properties such as its periodicity, symmetry or its chaotic nature in general [24]. Having now these new sequences as states, one can examine their statistics and compute the entropy, in a manner similar to the one proposed by Shannon, and thus draw useful conclusions about the characterization of the dynamics of nonlinear systems [25].

As a simple example, one can consider a time series of some real valued variable and perform a simple discretization by replacing the values in the series by either 1, if the value in question lies above some threshold *t*, and 0 if it is ≤t in case of binary partitioning. This “symbolic sequence” can be read in terms of distinct consecutive blocks of length *n*. For example, using n=2, one could obtain a sequence of patterns such as 01, 10, 10, 11, 00 and so forth, in a reading procedure that is called “lumping” or alternatively, these blocks could be read in a way that they overlap by one symbol at each time, hence producing the sequence of patterns 01, 11, 10, 01, 10, 01, 11 10, 00, and so forth, in a procedure called “gliding”. Following either of these two processes and counting the number of times that each pattern manifests will yield the probabilities of each, which can then be used to compute the entropy of the system. Typically, gliding is most commonly used as it produces more instances of blocks and thus better statistics, while lumping is important for the detection of automaticity, in series that have been generated by finite automata or algorithmic processes [26].

Extending Shannon’s classical definition of the entropy of a single state to the entropy of a succession of states [27,28], one reaches the definition of the n−block entropy, H(n), which is a measure of uncertainty and gives the average amount of information necessary to predict a subsequence of length *n*. Consequently, H(n)/n can be interpreted as the mean uncertainty per symbol and should converge for n→∞ to some stationary value if the observed dynamics is deterministic. Moreover, from a practical perspective, one is often interested in quantifying the mean information gain when increasing the word length, measured by the conditional (or differential) block entropies:

(2) $$\begin{array}{c} h(n)=H(n+1)-H(n)\;for\;n\geq 1;h(0)=H(1). \end{array}$$

For stationary and ergodic processes, the limit of h(n) for n→∞ provides an estimator of the Kolmogorov-Sinai entropy or source entropy *h* of the dynamical system under study [29,30] (in a similar spirit, H(n)/n provides another estimate of the source entropy for n→∞).

### 2.3. Alternative Entropy Formulations

Methodologies may vary, but the one thing that has remained constant so far has been the formulation of the entropy itself, that is, for the final calculations, the operation that was used entailed the logarithm of the probability (or average number of appearance) of certain events, according to the classical Boltzmann formalism. Nevertheless, alternative definitions of the same concept have existed prior to Shannon, such as the well known Hartley function introduced by Hartley in 1928 [31] and many more were proposed soon afterwards, as the one developed by Alfréd Rényi [32], that incorporates a free parameter which, when given certain values, can take the form of either Hartley or Shannon entropy and can thus be considered a more general form of both.

Based on the same mathematical reasoning, but also inspired by multi-fractal concepts, Tsallis introduced a similar expression, characterized by the index *q*, which in the framework of statistical physics can be used to extend the concept of entropy for systems that are not in thermodynamical equilibrium [33]. The formula for the Tsallis Entropy is:

(3) $$\begin{array}{c} S_{q}=k\frac{1}{q-1}\left(1-\sum p_{i}^{q}\right), \end{array}$$

with *k* being the corresponding Boltzmann constant. The parameter *q* determines the type and degree of non-extensivity of the system, that is, the divergence from statistical equilibrium. In normal, extensive systems, the entropy increases linearly with respect to the size of the system, for example, one dice has 6 equiprobable outcomes and thus an entropy value of log(6), while two dice yield $6^{2}$ outcomes and thus have an entropy of $\log\left(6^{2}\right)=2\log\left(6\right)$, that is, twice as large. The entropy of non-extensive systems can increase either faster than linearly or slower, reflecting the nature of the correlation between subsystems, in two cases that can thus be described as either super-additivity or sub-additivity. This is better depicted by the following rule, which determines the entropy of a system depending on the entropies of its two sub-parts:

(4) $$\begin{array}{c} S_{q}\left(A+B\right)=S_{q}\left(A\right)+S_{q}\left(B\right)+\left(1-q\right)S_{q}\left(A\right)S_{q}\left(B\right). \end{array}$$

In addition to this, the *q* parameter can also be seen as a “weighting factor” of the probabilities that are used for the calculation of the entropy, in a manner similar to the use of the free parameter of the Rényi Entropy. Given that $p_{i}$ will always be less than one, raising it to a power q>1 leads to the small probabilities (rare outcomes) becoming much more downgraded than high probabilities and thus, more common outcomes get treated preferentially. The opposite happens for q<1, in which case rare events are weighted more favourably, while for q=1 the formula converges to the typical Shannon Entropy.

All these formulations can be inserted in the definitions of entropies for the methodologies that were described above and by tweaking the free parameters of them, one can modify the results and thus focus on different parts of the dynamics of the system that is being examined (see also the special issue on Tsallis entropy published in Entropy [34]).

### 2.4. Approximate Entropy

Entropy is the rate of information production for dynamical systems. Methods for estimation of the entropy of a system represented by a time series are not, however, well suited to analysis of the short and noisy data sets encountered in various studies (e.g., biological). Data sets with similar properties are also met in space physics studies. Therefore, measures like the ones following have been specially designed to overcome these problems.

In order to avoid the discretization issues altogether, but maintain the basic idea of examining a time series in sequences of patterns, Pincus introduced [35] the formulation of Approximate Entropy (ApEn). Specifically, ApEn examines time series by making use of distances between sequences of successive observations, for detecting the presence of similar “epochs”; more similar and more frequent epochs lead to lower values of ApEn, while sequences of dissimilar epochs are interpreted as more randomness in the signal and thus result to higher entropy values. For a time series *s*, we can define N−m+1 vectors, each one consisting of *m* consecutive samples of this time series as:

(5) $$\begin{array}{c} X_{i}^{m}=\left\{s_{i},s_{i+1},s_{i+2},\ldots ,s_{i+m-1}\right\},i=1,2,\ldots ,N-m+1. \end{array}$$

The main idea is to consider a window of length *m* running through the time series and forming the corresponding vectors $X_{i}^{m}$. The similarity between the formed vectors is used as a measure of the degree of organization of the time series. A quantitative measure of this similarity $C_{i}^{m}\left(r\right)$, is given by the average number of vectors $X_{j}^{m}$, within a distance *r* from $X_{i}^{m}$. Here, $X_{j}^{m}$ is considered to be within a distance *r* from $X_{i}^{m}$ if $d_{ij}^{m}\leq r$, where $d_{ij}^{m}$ is the maximum absolute difference of the corresponding scalar components of $X_{i}^{m}$ and $X_{j}^{m}$ (i.e., the two vectors have a distance smaller than *r* according to their supremum norm). By calculating $C_{i}^{m}\left(r\right)$ for each i≤N−m+1 and then taking the mean value of the corresponding natural logarithms:

(6) $$\begin{array}{c} \phi^{m}\left(r\right)={\left(N-m+1\right)}^{-1}\sum_{i=1}^{N-m+1}\ln C_{i}^{m}\left(r\right), \end{array}$$

the ApEn is defined as:

(7) $$\begin{array}{c} ApEn\left(m,r\right)=\lim_{N\to \infty }\left[\phi^{m}\left(r\right)-\phi^{m+1}\left(r\right)\right], \end{array}$$

which, for a finite time series, can be estimated by the statistic:

(8) $$\begin{array}{c} ApEn\left(m,r,N\right)=\phi^{m}\left(r\right)-\phi^{m+1}\left(r\right). \end{array}$$

By tuning *r*, one can reach a reasonable degree of similarity for “most” vectors, $X_{i}^{m}$, and, hence, a reliable statistics, even for relatively short time series. In summary, the presence of repetitive patterns of fluctuation in a time series renders it more predictable than a time series in which such patterns are absent. A time series containing many repetitive patterns has a relatively small ApEn; a less predictable (i.e., more complex) process has a higher ApEn.

### 2.5. Sample Entropy

One of the issues that was raised in criticism of ApEn was the fact that its formalism does not exclude self-counts, namely that each vector of length ‘*m*’ will always find at least one matching vector, for any tolerance, that one match being of course the vector itself. To account for that bias and exclude self-matches, Richman and Moorman proposed a slightly modified measure called the Sample Entropy [36]. Sample Entropy (SampEn) follows the same rationale, but takes measures in order to ignore self-matches, that is, the search for similar vectors only happens for i≠j. Though, this way allows for the emergence of zero values in the counts of matched vectors, then ruling out the use of logarithms. Besides that, the entire formulation is updated to make sure that the search for similar patterns, for both ‘*m*’ as well as ’m+1’ vector lengths, is performed in the same subset of the series (note that in the case of ApEn the search for ‘*m*’ length vectors extends from the 1st up to element N−m+1, which means that for the next value of ‘*m*’ the series is reduced by 1 and it extends up to element N−(m+1)+1=N−m). In this manner, the previously defined $C_{i}^{m}\left(r\right)$ are now replaced by $B_{i}^{m}\left(r\right)$, defined as the ${\left(N-m-1\right)}^{-1}$ times the number of vectors of $X_{j}^{m}$ within distance *r* from $X_{i}^{m}$, and $A_{i}^{m}\left(r\right)$, which are defined exactly the same but for vector lengths m+1, that is, as the ${\left(N-m-1\right)}^{-1}$ times the number of vectors of $X_{j}^{m+1}$ within distance *r* from $X_{i}^{m+1}$. Hence, the formulas are now updated as:

(9) $$\begin{array}{c} B^{m}\left(r\right)={\left(N-m\right)}^{-1}\sum_{i=1}^{N-m}B_{i}^{m}\left(r\right), \end{array}$$

(10) $$\begin{array}{c} A^{m}\left(r\right)={\left(N-m\right)}^{-1}\sum_{i=1}^{N-m}A_{i}^{m}\left(r\right), \end{array}$$

and the SampEn is then defined as:

(11) $$\begin{array}{c} SampEn\left(m,r\right)=\lim_{N\to \infty }\left[-\ln\left[A^{m}\left(r\right)/B^{m}\left(r\right)\right]\right], \end{array}$$

which, for finite series is estimated by the statistic:

(12) $$\begin{array}{c} SampEn\left(m,r\right)=-\ln\left[A^{m}\left(r\right)/B^{m}\left(r\right)\right]. \end{array}$$

The quantity $A^{m}\left(r\right)/B^{m}\left(r\right)$ expresses the probability that two sequences within a tolerance of *r* for *m* points shall remain within the same tolerance of each other for m+1 points. Thus, the SampEn attempts to be a proxy of the source entropy of the underlying dynamic system, in the same spirit as the usage of the h(n)=H(n+1)−H(n) in the case of the block entropies.

### 2.6. Fuzzy Entropy

Expanding upon the concepts already established with ApEn and SampEn, Chen and his colleagues combined elements from Fuzzy Sets and Information Theory to develop a fuzzy version of the Sample Entropy [37]. Their approach introduces two major elements. The first, that each $X_{i}^{m}$ vector is now transformed by the subtraction of a baseline that is equal to the mean value of its elements, that is,

(13) $$\begin{array}{c} X_{i}^{m}=\left\{s_{i},s_{i+1},s_{i+2},\ldots ,s_{i+m-1}\right\}-m^{-1}\sum_{j=1}^{m-1}s_{i+j}, \end{array}$$

which now allows comparisons between vectors to be performed not in the sense of their actual proximity, but rather in a more “dynamic” way, based on whether they exhibit for example, increasing or decreasing values relative to their average mid-point.

The second idea, which is the one influenced by Fuzzy Set theory, is to replace the distance based count of matching patterns with a Fuzzy membership function. In this way, two vectors are not considered a match only if their distance (again in the supremum norm sense) is lower than a certain threshold, but to each pair is assigned a real value, ranging from 0 to 1, that attempts to quantify exactly how much these two vectors are considered similar to one another. Many such functions exist in the literature, but for the purposes of this work the simple Gaussian membership function was implemented, which yields the value of 1 if two vectors are exactly identical (distance equal to 0) and values less than 1 for increasing distances, according to the formula:

(14) $$\begin{array}{c} D_{i,j}^{m}=\exp\left(-{\left(d_{i,j}^{m}\right)}^{2}/\left(2r^{2}\right)\right), \end{array}$$

where $d_{i,j}^{m}$ is again the supremum norm of the two vectors and *r*, the previously defined distance threshold, is now used as the sigma value of the Gaussian function. Due to this, the algorithm does not simply count the number of matches for a specific pattern, but rather it sums the values of the output of the membership function, introducing a “fuzziness” element in the process. The rest of the method is exactly the same as in the case of SampEn so the formalism is as follows:

(15) $$\begin{array}{c} \phi_{i}^{m}\left(r\right)={\left(N-m-1\right)}^{-1}\sum_{i=1,i\neq j}^{N-m}D_{i,j}^{m}\left(r\right), \end{array}$$

(16) $$\begin{array}{c} \phi^{m}\left(r\right)={\left(N-m\right)}^{-1}\sum_{i=1}^{N-m}\phi_{i}^{m}\left(r\right), \end{array}$$

(17) $$\begin{array}{c} \phi^{m+1}\left(r\right)={\left(N-m\right)}^{-1}\sum_{i=1}^{N-m}\phi_{i}^{m+1}\left(r\right), \end{array}$$

and finally the Fuzzy Entropy itself is being defined by:

(18) $$\begin{array}{c} FuzzyEn\left(m,r\right)=\lim_{N\to \infty }\left[\ln\left[\phi^{m}\left(r\right)/\phi^{m+1}\left(r\right)\right]\right], \end{array}$$

which, for finite series is estimated by the statistic:

(19) $$\begin{array}{c} FuzzyEn\left(m,r\right)=\ln\left[\phi^{m}\left(r\right)/\phi^{m+1}\left(r\right)\right]. \end{array}$$

To summarize:

- The uncertainty of an open system state can be quantified by the Boltzmann-Gibbs (B-G) entropy, which is the widest known uncertainty measure in statistical mechanics. B-G entropy cannot, however, describe nonequilibrium physical systems characterized by long-range interactions or long-term memory or being of a multi-fractal nature. Inspired by multi-fractal concepts, Tsallis [38,39] has proposed a generalization of the B-G statistics, that is, the Tsallis Entropy, $S_{q}$.
- Approximate entropy (ApEn) has been introduced by Pincus as a measure for characterizing the regularity in relatively short and potentially noisy data. More specifically, ApEn examines time series for detecting the presence of similar epochs; more similar and more frequent epochs lead to lower values of ApEn.
- Sample entropy (SampEn) was proposed by Richman and Moorman as an alternative that would provide an improvement of the intrinsic bias of ApEn.
- Fuzzy entropy (FuzzyEn), like its ancestors, ApEn and SampEn, is a “regularity statistic” that quantifies the (un)predictability of fluctuations in a time series. For the calculation of FuzzyEn, the similarity between vectors is defined based on fuzzy membership functions and the vectors’ shapes. FuzzyEn can be considered as an upgraded alternative of SampEn (and ApEn) for the evaluation of complexity, especially for short time series contaminated by noise.

## 3. Data and Analysis

### 3.1. Swarm Magnetic Field Data

The Swarm mission [40] is the fourth Earth Explorer of the European Space Agency (ESA), launched on 23 November 2013. Swarm is a constellation of three satellites with two spacecraft (Swarm A and C) flying side by side at low altitude (∼460 km) and one third spacecraft (Swarm B) flying at a slightly higher altitude (∼510 km). All three satellites were launched at low-Earth near-polar orbit with the aim to provide the most accurate survey of the Earth’s magnetic field and its variations with time, including the Sun’s influence on the Earth system by analyzing electric currents in the magnetosphere and ionosphere and understanding the impact of solar wind on the dynamics of the upper atmosphere.

For the context of this study, the Magnetic Level 1b product was used, containing data from the Vector Field Magnetometer (VFM) at 1 Hz resolution. Data, along with the rest of the Swarm mission products, are available at the website ftp://swarm-diss.eo.esa.int upon registration. The CHAOS-6 Geomagnetic Field [41] was used to remove the influence of the terrestrial internal magnetic field, that is, the field generated by the Earth’s liquid outer core and the field trapped in the planet’s crust, so that what remains is the variations of the magnetic field due to ionospheric and magnetospheric current systems, which are directly or indirectly influenced by the solar wind conditions. Lastly, the total magnitude of the remaining vector field was computed, in units of nT.

Experimental data are usually affected by erroneous measurements, that typically appear in the form of spikes in the data series, so an intermediate data cleaning step was included. Such cleaning can be performed in many ways, but due to the obvious nature of the outliers in this case, a simple threshold-based removal process was deemed enough. To set the threshold, instead of the typical variance or standard deviation measures that are normally used, it was opted to design a “variation” measure based on percentiles, which are far less affected by the presence of large-valued spikes and, thus, are more robust in the presence of such noise. Following the motivation behind the well-established Interquartile Range (IQR), which is the difference in the values of the 75th and 25th percentile of a given dataset [42], a similar measure (IPR90) defined as the difference between the 90th and 10th percentile of the data was designed and used to identify as outliers all points that exceeded the median series by a certain number of times of this new inter-percentile range, that is,

(20) $$\begin{array}{c} x_{i}\in outliers\;if\;\left|x_{i}-median\left(x\right)\right|>k\cdot IPR90, \end{array}$$

with *k* being a free parameter typically accepting values around 10 for the very obvious cases of outliers (as was this study) or values of about 2 or 3 on a moving window of about 20 points for the more elusive ones.

**Figure 1:**
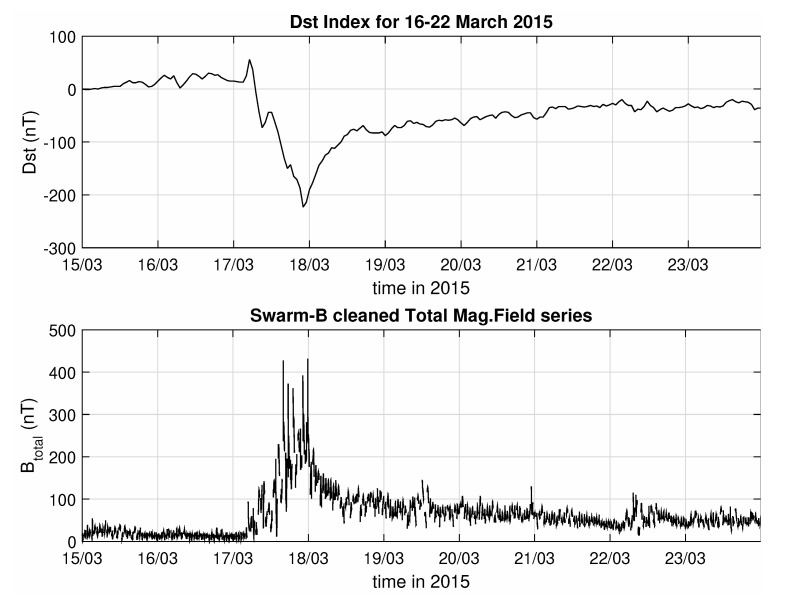
Comparison between the Dst index (**top panel**) and the pre-processed series of the total (external) magnetic field (**bottom panel**), as measured by Swarm B, during the March 2015 storm.

The purpose of the analysis is to focus on the effect of magnetic storm’s on the Earth’s field, an effect that leads to the enhancement of the ring current system, an equatorial, toroidal current of energetic charged particles, which is responsible for global decreases in the Earth’s surface magnetic field during storms [43]. To focus on these variations of the magnetic field, only measurements recorded between magnetic latitudes from −50° to +50° were considered, while the rest were discarded. An example of the resulting time series from Swarm B, for the period covering the magnetic storm of March 2015, is shown in Figure 1, where it is evident how close the pre-processed data follow the evolution of the Dst index and thus of the storm itself.

**Figure 2:**
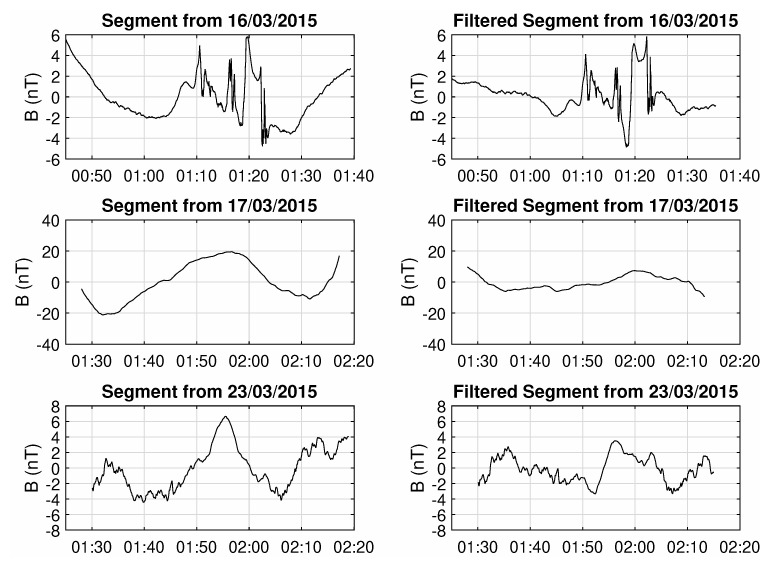
Example of three segments before (**left**) and after filtering (**right**), for the pre-storm phase (**top row**), the peak of the storm (**middle row**) and after the end of event (**bottom row**), respectively.

Of course, removing the high-latitudinal data where the satellite passes above the poles, breaks the continuous time series to an ensemble of isolated segments of equatorial crossings, composed of approximately 1400 points (23.3 min) and thus the analysis needs to be performed on a segment-by-segment basis. Since the system needs to be viewed as an information generator in order for the measures outlined in Section 2 to be applicable, the absolute value of the points in each segment is not really important. What is important is the degree of disorder that is captured by the variations of the magnetic field and thus, the mean value of each segment is being subtracted from the data. Additionally, to remove the natural oscillations in the field measurements as the satellite flies from negative mid-latitudes (higher field intensity) to the equator (lowest field) and then to positive mid-latitudes (higher field), a two-minute moving average filter was derived and subtracted from the segment data. Three characteristic examples are shown in Figure 2, where a segment from before the onset of the storm and one from long after the end of the event can be compared to a typical segment from the near-peak of the storm’s activity. As can be seen, the first and last segments show a much higher degree of randomness, observable by the “roughness” of their series, indicating that the state of the system is closer to that of random noise. In contrast, segments that were taken during the main phase (or even the beginning of the recovery) exhibit much more organized behaviour, resulting in smoother series, without many sudden variations or similar anomalies, indicating that the system is now in a more well-coordinated phase, typical of large scale correlations and critical behaviour.

### 3.2. Entropy Analysis of the Swarm B Magnetic Field Data for the St. Patrick’s 2015 Storm

On 17 March 2015, the Dst index captured the strongest magnetic storm of solar cycle 24 (the St. Patrick’s Day storm with a minimum Dst index of −223 nT). De Michelis et al. [44] used Swarm data to study the properties of the magnetic field’s fluctuations produced by ionospheric and magnetospheric electric currents during this storm. They examined the different latitudinal structure of the geomagnetic field fluctuations and analysed the dynamical changes in the magnetic field scaling features during the development of the storm. Analysis revealed consistent patterns in the scaling properties of magnetic fluctuations and striking changes between the situation before the storm, during the main phase and the recovery phase. They discussed these dynamical changes in relation to those of the overall ionospheric polar convection and potential structures as reconstructed using SuperDARN data. Their findings suggested that distinct turbulent regimes characterised the mesoscale magnetic field’s fluctuations and that some factors, which are known to influence large-scale fluctuations, had also an influence on mesoscale fluctuations. Other studies of the dynamics of the 2015 St. Patrick’s Day storm can be found in References [45,46].

Now having clean, pre-processed and filtered segments of this type at hand, it is easy to perform an entropy analysis, using as a starting point the formulation of the well-known Shannon Entropy. The entropic analysis can be performed in two ways—firstly, using the typical Histogram Entropy, by defining 20 bins with values between the minimum and maximum of a certain segment, counting the number of measurements that fall within each such bin and computing the entropy and secondly, using the Block Entropy approach, for both Lumping and Gliding methods in a binary digitization of the segment values, producing the value for the Shannon Block Entropies for block lengths of for example, up to 3 points and computing the slope of H(n)/n as a proxy of the source entropy *h*. This approach has been already applied in the past [18,47] to study the Dst index using windows of 128 or 256 hourly points (i.e., windows of 5 or 10 days in length). In our case the same approach applied to the 1-sec Swarm data provides a unique opportunity to examine shorter time scales. Hence, the analysis was performed on each segment individually and then daily averages of the procured values were computed, in order to capture enough of the dynamic behaviour reflected in the data, but also to be able to follow the changes of this behaviour for the various phases of the storm, in a manner similar to the one used in the previous study.

**Figure 3:**
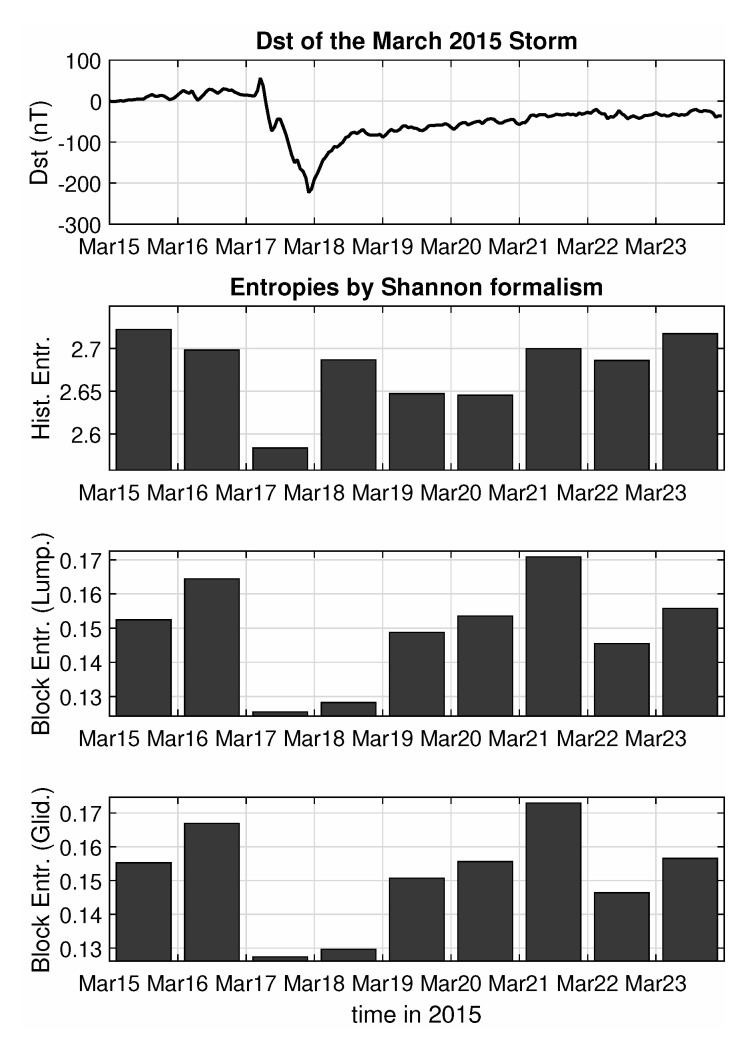
Entropy analysis according to Shannon formalism of the Swarm B total (external) field for the March 2015 magnetic storm.

As can be seen from the results of the analysis, plotted in Figure 3, both Block Entropies show a sharp decrease at the two days of peak activity (main phase and beginning of recovery) compared to the high entropic values before the event, showcasing the change in the level of complexity in the system, from highly disorganized (random noise) to highly organized (critical behaviour). The entropy levels return to their high pre-storm values, a few days after the event, but then exhibit a second, although not as pronounced, local minimum at 22 March, which is also visible in the Dst index series, as for this day the index also exhibits a small drop to about −40 nT. This is probably related to sub-storm activity in the nightside, as the AL index [48], which is typically used to describe substorms [49], also shows a sudden drop in its values for the same day. Unfortunately, the simple histogram entropy only captures the 17 March drop, but then fails to depict a clear picture and so cannot be considered as an appropriate measure for the purposes of this study.

**Figure 4:**
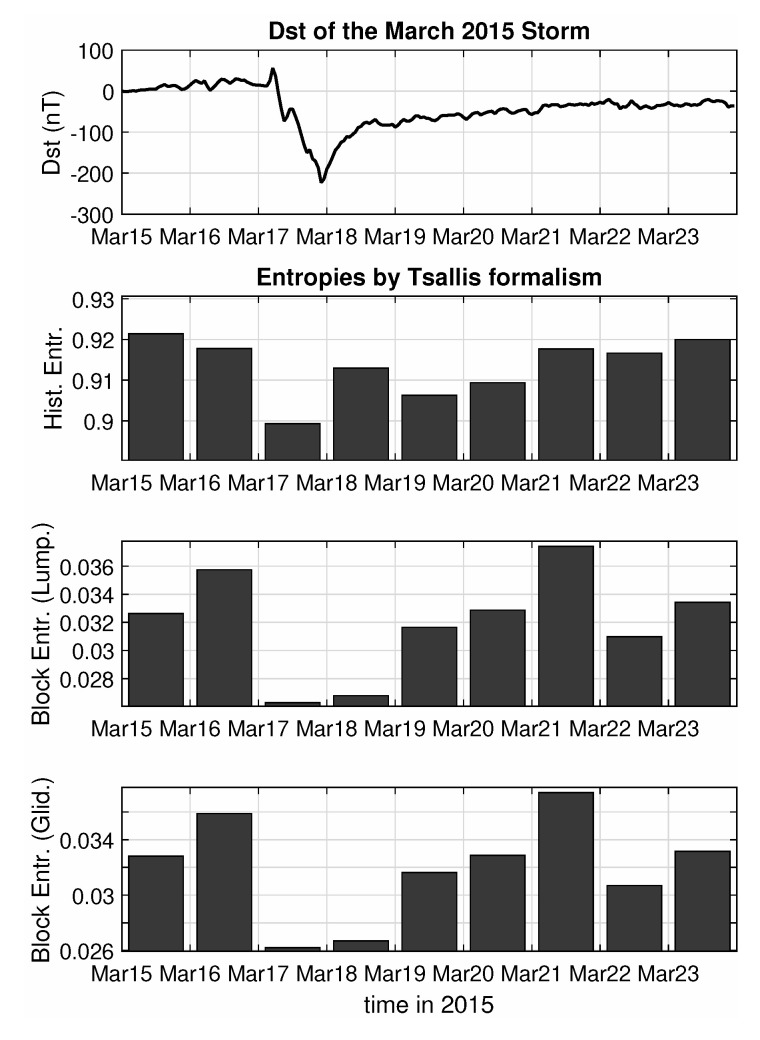
Entropy analysis according to Tsallis formalism of the Swarm B total (external) field for the March 2015 magnetic storm.

Similar results can be drawn by repeating the analysis with the other entropy formulation, as can be shown in Figure 4 that follows, for the cases of the Tsallis Entropy, for a free parameter value of 2. It should be noted that the small numbers produced by the Tsallis formalism do not mean zero entropy, but are a side effect of using exponent values q>1, while the relative drop of entropy for the peak of the storm is actually higher in this case compared to the results with the Shannon formula. The image remains the same for other q>1 values, while a choice of q<1 yields similar results qualitatively, but with less accentuated differences for the two days of the peak of the storm and the secondary minimum at March 22.

The same picture can be produced by performing the analysis using the framework of Approximate, Sample and Fuzzy entropies. The free parameter is now the distance threshold that will be used to identify similar or distinct blocks, while there is no symbolic representation (binary or otherwise) but rather the values in the segment’s series are used as they already are. This distance threshold is usually expressed as a multiple of the standard deviation of the values in the examined segment, and after some experimentation, the final values were set to 0.1·st.dev for ApEn and SampEn and 1·st.dev for FuzzyEn, since the last one uses a more “soft threshold” compared to the former two.

**Figure 5:**
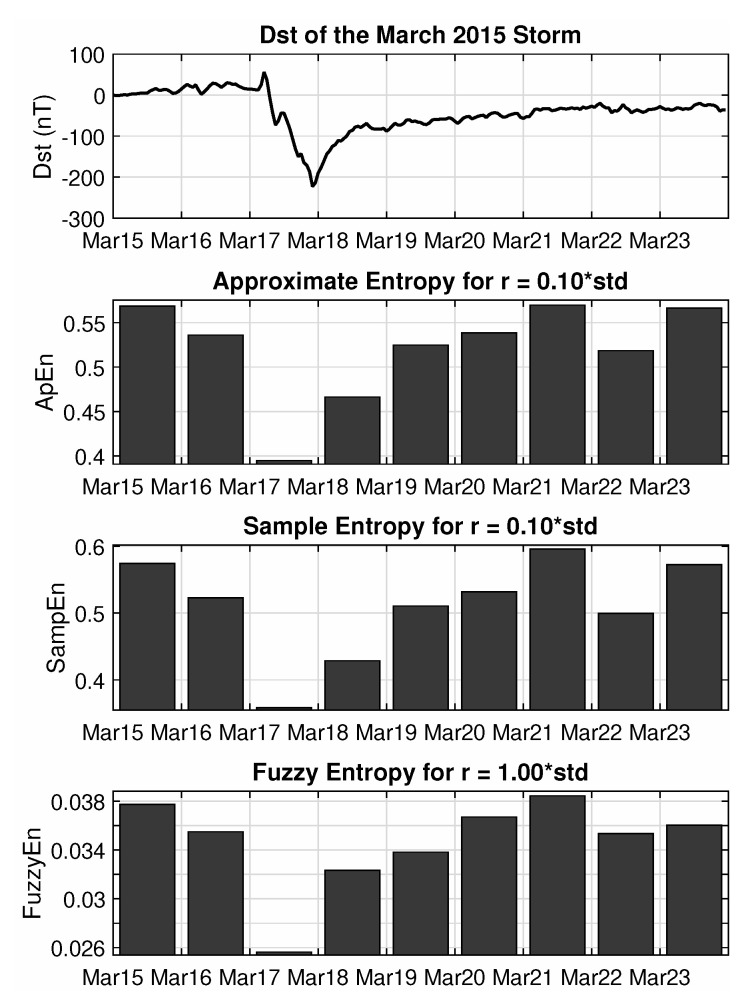
Approximate, Sample and Fuzzy entropy analysis of the Swarm B total (external) magnetic field for the March 2015 magnetic storm.

The output, as seen in Figure 5, again shows high entropic values before the onset of the storm, accompanied by a rapid decrease, for the peak of the minimal Dst value, which are then followed by a slow and gradual increase of the levels of randomness at the recovery phase of the storm. The local minimum on 22 March is likewise observed in the results of this analysis as well, also indicating the presence of some follow-up activity, albeit of lesser impact. There are though some systematic differences between these results and the ones procured by the previous measures, especially for the first day of the studied time period, which shows higher entropy values for ApEn, SampEn and FuzzyEn compared to the Block Entropies. Looking closely back at the bottom panel of Figure 1, it can be argued that even though both the first and second days appear as random noise series, the first one has higher variance. This is something that is ignored by the symbolic dynamics approach, which just uses a binary representation, but is taken seriously into account by the ApEn, SampEn and FuzzyEn, which all use the actual values to decide if two blocks are similar enough to be counted as one or not. Thus, blocks that would appear identical for the Block Entropies, might appear as different for ApEn and its relative entropies, resulting in more occurrences of different patterns and as such, in higher entropy values. Due to a similar line of reasoning, the second family of entropies show higher values also for the second day of the event (18 March), depicting a much faster recovery to pre-storm entropy levels, whereas Block Entropies are slower in registering these changes, describing a much slower recovery process.

## 4. Conclusions and Discussion

This study shows how viewing the magnetosphere as an information generating system can yield valuable insights about the onset and evolution of magnetic storms. Although previous studies have also produced similar results, this is the first time that satellite data from a LEO mission have been used in this manner, proving that if effectively treated they can not only replicate past conclusions, but also present a unique opportunity to revisit the same phenomena in much faster time scales, utilizing the 1 s sampling time of Swarm measurements in contrast to the 1 min or 1 h resolution of most indices of geomagnetic activity. Furthermore, Swarm in situ observations may offer an unprecedented view of the ionosphere response to extreme events in geospace like magnetic storms [46]. Extreme magnetic storms may lead to extremes in ionospheric behavior with consequences for a variety of technological systems [50].

In addition to that, sophisticated entropy-based methods such as ApEn or FuzzyEn or measures that make use of the concepts of symbolic dynamics, can accurately capture the changes of the various dynamic states of the system, from the main phase to the recovery period, and are also sensitive enough to highlight cases where similar changes take place, even though they are much less pronounced in the time series. The substorm activity of 22 March is a characteristic example of that, since it produces a drop in entropy that is about half of the drop that is observed for the peak of the storm, even though its intensity in the time series is less than a quarter of the peak, as can be seen in Figure 1. This becomes even more important considering that the methodology removes every information about the absolute value of the data points, during the filtering step and even more so for the applications of symbolic dynamic entropies, which convert all values to either 1 s or 0 s, yet are still able to discern that the system has now passed to another stage regarding its degree of organization or complexity. Thus, entropy analysis successfully differentiates between the quiet-time and storm-time magnetosphere and highlights the transition of the magnetosphere-ionosphere-thermosphere (MIT) coupling system to a more ordered state with higher organization or less complexity associated with the occurrence of the extreme geospace event.

It is our strong belief that such tools can be used to characterize the state of the MIT coupling system and thus be used to help measure its response to subsequent excitation by the incoming solar wind, effectively enhancing ongoing nowcasting or forecasting activities.

For future studies, we plan to expand our entropic analysis and perform it to more diverse and complex datasets perhaps from ground based measurements of the Earth’s magnetic fields, for example the family of the AE indices. Trying to understand the contributors to the magnetic field variations, that is, the spatial locations and temporal variations of the different current systems in the ionosphere and magnetosphere, will be useful to space scientists.
